# High-dose short-term creatine supplementation without beneficial effects in professional cyclists: a randomized controlled trial

**DOI:** 10.1080/15502783.2024.2340574

**Published:** 2024-04-12

**Authors:** David Barranco-Gil, Lidia B. Alejo, Carlos Revuelta, Miguel Górriz, Itziar Pagola, Laureano M. Ozcoidi, Alejandro Lucia, Pedro L. Valenzuela

**Affiliations:** a Universidad Europea de Madrid, Faculty of Sport Sciences, Madrid, Spain; b Research Institute of Hospital 12 de Octubre (imas12), Physical Activity and Health Research Group (PAHERG), Madrid, Spain; c Caja Rural Professional Team, Navarra, Spain; d University of Alcalá, Department of Systems Biology, Madrid, Spain

**Keywords:** Recovery, performance, nutrition, cycling, supplement

## Abstract

**Background:**

Growing evidence supports the ergogenic effects of creatine supplementation on muscle power/strength, but its effects on endurance performance remain unclear. We assessed the effects of high-dose short-term creatine supplementation in professional cyclists during a training camp.

**Methods:**

The study followed a double-blind, randomized parallel design. Twenty-three professional U23 cyclists (19 ± 1 years, maximum oxygen uptake: 73.0 ± 4.6 mL/kg/min) participated in a 6-day training camp. Participants were randomized to consume daily either a recovery drink (containing carbohydrates and protein) with a 20-g creatine supplement (creatine group, *n* = 11) or just the recovery drink (placebo group, *n* = 12). Training loads and dietary intake were monitored, and indicators of fatigue/recovery (Hooper index, countermovement jump height), body composition, and performance (10-second sprint, 3-, 6-, and 12-minute time trials, respectively, as well as critical power and W’) were assessed as study outcomes.

**Results:**

The training camp resulted in a significant (*p* < 0.001) increase of training loads (+50% for total training time and + 61% for training stress score, compared with the preceding month) that in turn induced an increase in fatigue indicators (significant time effect [*p* < 0.001] for delayed-onset muscle soreness, fatigue, and total Hooper index) and a decrease in performance (significant time effect [*p* = 0.020] for critical power, which decreased by −3.8%). However, no significant group-by-time interaction effect was found for any of the study outcomes (all *p* > 0.05).

**Conclusions:**

High-dose short-term creatine supplementation seems to exert no consistent beneficial effects on recovery, body composition or performance indicators during a strenuous training period in professional cyclists.

## 1. Introduction

Creatine is among the few dietary supplements with a documented ergogenic effect [1,2]. Indeed, high-dose short-term supplementation (e.g. 20 g for 5–7 days) effectively increases the availability of creatine and phosphocreatine in skeletal muscle and brain [3,4], which has important implications on ATP production in these tissues, among other functions (e.g. antioxidant capacity). Meta-analytical evidence shows in fact that creatine supplementation enhances performance in different muscle strength/power indicators, particularly during short-duration (<3 min) efforts [5,6]. Moreover, despite a few studies finding no significant benefits [7,8], strong evidence – including a meta-analysis of 14 randomized controlled trials – shows that short-term creatine supplementation is an effective strategy for improving muscle strength, fatigue resistance or power output (PO) during a single supramaximal effort (e.g. 100-m running sprint, Wingate anaerobic test) or repeated sprints [9–17].

Despite the abovementioned evidence, the effects of creatine supplementation in the context of endurance sports remain largely unexplored. A recent meta-analysis found that creatine supplementation exerts no significant beneficial effects on endurance performance as assessed with ‘classic’ indicators such as the lactate threshold, maximum oxygen uptake (VO_2max_), time trial (TT) performance, or time to exhaustion [18]. However, given its potential to facilitate ATP resynthesis while also buffering hydrogen ion accumulation, it has been recently hypothesized that, despite potential increases in body mass, creatine supplementation might be effective to improve endurance performance, particularly in those endurance sports involving repeated high-intensity efforts [19]. For instance, there is preliminary evidence for a potential ergogenic effect in repeated cycling sprints [20] or in the closing sprints of an exhaustive cycling TT [21]. On the other hand, creatine supplementation might enhance recovery in endurance sports [19] through an attenuation of inflammation and oxidative stress [21,22] or by reducing delayed-onset muscle soreness (DOMS) [23], as well as through improvements in glycogen resynthesis when co-ingested with carbohydrates [21,22].

The abovementioned benefits on recovery and performance could be of special interest during periods of repeated strenuous training loads, such as those undergone by cyclists during multi-stage races or training camps. Moreover, it would be interesting to assess the effects of creatine supplementation not only on short-duration high-intensity efforts (e.g. sprint ability), but also on key markers of endurance performance such as critical power (CP) [24,25]. CP has been reported to represent the gold standard measure of the highest constant work rate that can be sustained via a steady state of substrate utilization and resynthesis while preserving physiological homeostasis (i.e. without depletion of muscle high-energy phosphates or rapid accumulation of hydrogen ions and inorganic phosphate in muscle tissue or in the bloodstream) [24–26]. Thus, the CP is usually considered as the boundary between steady state and non-steady state exercise conditions, or between heavy and severe exercise domains [24,25]. However, to the best of our knowledge the effects of creatine supplementation on CP remain unknown.

The aim of this study was to analyze the effects of high-dose, short-term creatine supplementation on recovery/fatigue indicators, body composition, and field-based performance in professional cyclists during a training camp. We hypothesized that this nutritional intervention might improve recovery and endurance performance, at least during short-duration efforts.

## 2. Material and methods

### 2.1. Participants

Twenty-three professional U23 cyclists (19 ± 1 years) from the same team volunteered to participate in the study. They all competed at national or international level. To be enrolled in the study, cyclists had to be free of musculoskeletal injuries or other conditions that could hinder their participation. None of them was taking any type of medication or dietary supplement (other than the creatine or placebo supplement as per group assignment) and they were instructed to maintain their usual diet (e.g. usually composed of 60% carbohydrate, 25% protein and 15% fat) during the study period.

Participants’ VO_2max_ (73.0 ± 4.6 mL/kg/min) and peak PO (PPO, 6.9 ± 0.4 W/kg) were determined as descriptive variables in our laboratory following the procedures explained elsewhere [27]. Briefly, the cyclists performed a graded test (25 W/min) on their own bikes, which were placed on a validated indoor trainer (Hammer, CycleOps, Madison, WI) [28], and gas exchange data were collected breath-by-breath with a metabolic cart (Ultima Series Medgraphics; Cardiorespiratory Diagnostics, Saint Paul, MN). Attending to these variables, participants could be considered of the highest performance level (i.e. level 5) following the guidelines proposed by De Pauw et al. [29]. They were informed of the study procedures and provided written informed consent. The study was approved by the Ethical Committee of Alcorcón University Hospital (Madrid, Spain; approval number 20/201), and all procedures were conducted following the standards established by the Declaration of Helsinki and its later amendments.

### 2.2. Experimental design and nutritional intervention

**Figure 1.**
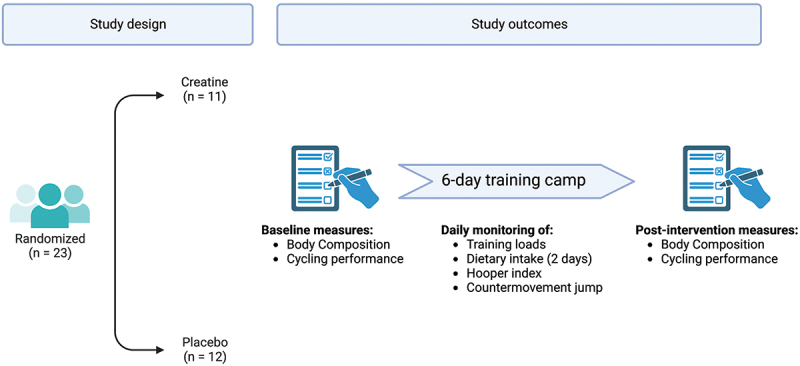
Schematic summary of the study design. Creatine (20 g/day) was administered for a total of 7 days, also including the day preceding the training camp.

The study followed a double-blind, parallel-group randomized controlled design. The study took place during a 6-day training camp at the end of the ‘pre-season’ period (January 2023), during which participants performed a training session every morning (~09.00 am to ~ 12.00–15.00 pm). The study design is summarized in Figure 1 and an example of training session is available as Supplementary File.

Participants were randomized by an external researcher using a specific software (https://www.sealedenvelope.com/) to either a ‘placebo’ (*n* = 12) or “creatine” group (*n* = 11). The day before the first training session and immediately after each session (therefore ingesting creatine for a total of 7 days) all participants ingested a recovery drink following the recommendations proposed elsewhere (i.e. 0.9 g/kg/h of carbohydrates and 0.3 g/kg/h of whey protein) [30]. In the creatine group, the recovery drink also contained 20 g of creatine monohydrate (Pure Creatine-100% Creapure, Victory Endurance, Weider; Madrid, Spain).

Researchers involved in supplement administration or outcome assessment were blinded to group assignment throughout the entire duration of the study. To ensure participants’ blinding, all recovery drinks were dispensed in identical opaque bottles, and were similar in appearance and taste.

### 2.3. Measurements

#### 2.3.1. Training load control

Daily training loads were monitored during the month preceding the study as well as during the training camp. PO data were registered during all training sessions with a power meter (Shimano Dura-Ace FCRC9100-P, Shimano; Sakia, Japan). Training volume (total time/distance covered), work (kJ spent), and the Training Stress Score (TSS) were also recorded for each session. TSS was computed as:

(1) $$\mathrm{TSS}\;=\;\left(\left(t*\mathrm{NP}*\mathrm{IF}\right)\;/\;\left(\mathrm{FTP}*3600\right)\right)\;*\;100$$

where *t* = duration (s), NP = normalized PO, and IF = intensity factor (NP/functional threshold power [FTP]). The FTP was calculated as 95% of the highest PO attained for a 20-minute effort during the previous 4 weeks [31]. Data from all the sessions was analyzed using a commercialized training analysis software (WKO5 Build 576; TrainingPeaks LLC; Boulder, CO).

#### 2.3.2. Nutritional assessment

Participants completed a 2-day (1 weekday and 1 weekend day) food report during the training camp and captured photos of all the food ingested during these 2 days. Nutritional intake was analyzed using a specific software (DietoPro; Valencia, Spain) and the mean intake of macronutrients during the 2 days was computed and averaged.

#### 2.3.3. Recovery and fatigue

The Hooper’s index–—a practical and valid indicator of athletes’ internal load [32]—was recorded every morning after waking up [32]. Participants rated their perceived sleep quality, stress, fatigue, and DOMS on a 7-point scale, with minimum ( = 1) and maximum scores ( = 7) representing “very, very good” and “very, very poor,” respectively. A total score was computed by summing the aforementioned 4 components.

Every morning before training, participants performed 3 countermovement jumps (CMJ) and the mean height was calculated using a contact platform (Chronojump, Boscosystem; Barcelona, Spain) [33]. They were instructed to perform a downward movement to reach approximately 90º of knee flexion while maintaining their hands on the hips. Cyclists were also instructed not to flex their knees during the flight in order to avoid overestimation of the flight time. Changes in jumping ability have been previously reported to be highly correlated to metabolic (i.e. increases in lactate and ammonia) and mechanic measures of fatigue [34].

#### 2.3.4. Body composition

Body mass and body composition were assessed the day before and after the training camp using bioelectrical impedance analysis (InBody 720, Inbody USA; Cerritos, CA) after at least 24 hours of rest, with participants instructed to attend the laboratory euhydrated and to empty their urinary bladder at least 30 minutes before assessment. This instrument has previously proven reliable for the measurement of body composition when more accurate methods (e.g. dual-energy X-ray absorptiometry) are not available [35–37]. Height was measured using a stadiometer (Harpender, Holtain Limited; Crymich, UK).

#### 2.3.5. Field-based performance

The day before and after the training camp (following body composition assessment), participants performed a field-based 10-second all-out sprint, and the mean PO and PPO registered during this test were used for analyzes.

Thirty minutes later, participants performed three separate field-based TT of 3, 6, and 12 minutes, respectively (consistently in this order) and the PO attained during each TT was registered. The TT were interspersed by 30 minutes of passive recovery, as done in previous studies [38–41]. No specific pacing strategy was recommended, although participants were encouraged to achieve the highest mean PO possible. All TT were performed on the same uphill climb (Haza Llana Pass, Andalucía, Spain: distance = 16.5 km, average slope = 5.45%) and at the same time of the day. Ambient temperature was measured with a portable meteorological station (Kestrel Meter 5500; Boothwyn, PA), and remained similar between testing sessions (15–16 ºC). Using the mean PO attained in the 3, 6 and 12-minute TT, critical power (CP) and W’ were calculated with the ‘1/t method’, as explained elsewhere [42].

### 2.4. Statistical analysis

Data are shown as mean ± standard deviation. The normal distribution (Shapiro – Wilk test) and homoscedasticity (Levene’s test) of the data was checked before any statistical treatment. Between-group differences in variables assessed at a single time point (e.g. baseline descriptive variables, dietary intake, training loads) were determined through Students’ unpaired *t*-tests, whereas differences in those variables assessed at several time points were determined with a mixed-model ANOVA, setting group and time as the between- and within-subject factors, respectively. In order to reduce the risk of type I error, post hoc differences (Bonferroni test) were only checked when a significant group by time interaction effect was found. Effect sizes (Cohen’s d) were computed for study outcomes. Analyses were performed using SPSS (version 23.0, IBM, NY) and the significance level was set at 0.05.

## 3. Results

The two groups showed similar characteristics at baseline (Table 1). Compared with the preceding month, the training camp induced a significant increase in training loads, as reflected by raises in total weekly training time (16.4 ± 1.7 versus 24.6 ± 1.1 hours, respectively, *p* < 0.001), load (TSS: 749 ± 100 versus 1203 ± 138 TSS, *p* < 0.001), distance (450 ± 55 versus 686 ± 34 km, *p* < 0.001) or work (10,545 ± 1,612 versus 15,365 ± 2,149 kJ, *p* < 0.001). No between-group differences were observed in training loads or in dietary intake during the training camp (Table 2).

**Table 1.** Descriptive characteristics of study participants by group.

Training load	*Creatine* *(n = 11)*	*Placebo* *(n = 12)*	P-value between groups	Cohen’s d
Age (years)	19 ± 1	19 ± 1	0.285	0.462
Body mass (kg)	65.8 ± 6.9	65.6 ± 6.0	0.951	0.030
BMI (kg/m^2^)	20.5 ± 1.6	20.6 ± 1.5	0.900	0.064
VO_2max_ (mL/kg/min)	72.3 ± 5.2	73.8 ± 4.0	0.452	0.323
PPO (W)	445 ± 29	459 ± 38	0.342	0.414
PPO (W/kg)	6.8 ± 0.4	7.0 ± 0.4	0.238	0.500

Data are shown as mean ± SD. Abbreviations: BMI, body mass index; PPO, peak power output, VO_2max_, maximum oxygen uptake.

**Table 2.** Training variables and daily dietary intake of study participants during the training camp.

Training load	*Creatine* *(n = 11)*	*Placebo* *(n = 12)*	P-value between groups	Cohen’s d
**Training variables**				
Time (hours)	24.5 ± 1.1	24.6 ± 1.0	0.906	0.095
Distance (km)	683 ± 35	689 ± 34	0.635	0.173
TSS (a.u.)	1,207 ± 150	1,201 ± 132	0.917	0.042
Work (kJ)	14,927 ± 2243	15,766 ± 2073	0.361	0.321
**Dietary intake**				
Total energy (kcal/day)	3,156 ± 235	3,221 ± 352	0.616	0.217
Carbohydrate (g/day)	368 ± 55	368 ± 43	0.976	0.000
Carbohydrate (g/kg/day)	5.6 ± 1.0	5.7 ± 0.8	0.988	0.010
Carbohydrate (%)	57.5 ± 4.4	57.3 ± 4.7	0.938	0.043
Protein (g/day)	153 ± 19	160 ± 14	0.345	0.419
Protein (g/kg/day)	2.4 ± 0.4	2.5 ± 0.4	0.487	0.297
Protein (%)	24.2 ± 3.0	25.0 ± 2.5	0.477	0.289
Fat (g/day)	116 ± 18	114 ± 31	0.881	0.078
Fat (g/kg/day)	1.8 ± 0.4	1.7 ± 0.5	0.822	0.090
Fat (%)	18.3 ± 3.5	17.7 ± 4.4	0.689	0.150

Data are shown as mean ± SD. Abbreviations: a.u., arbitrary units; TSS, training stress score.

### 3.1. Fatigue monitoring

**Figure 2.**
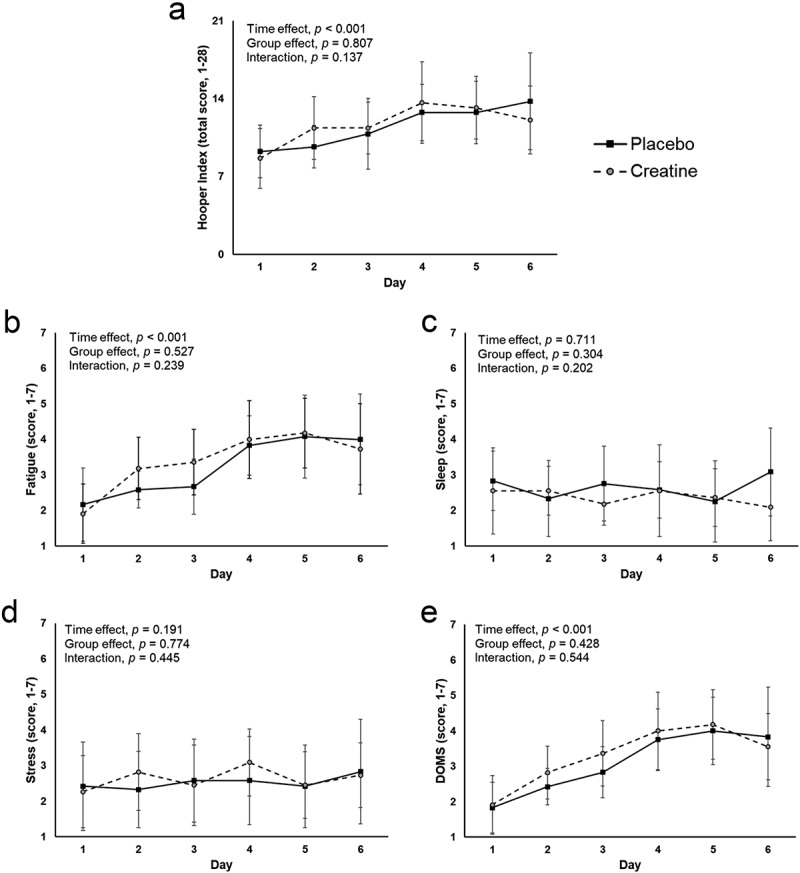
Differences between study groups for the Hooper index (panel A) and its sub-items (panels B-E) during the training camp.

A significant time and group interaction effect was found for CMJ, with higher values in the creatine group through the training camp (Figure 2). However, no significant group by time interaction effect was found for this outcome.

**Figure 3.**
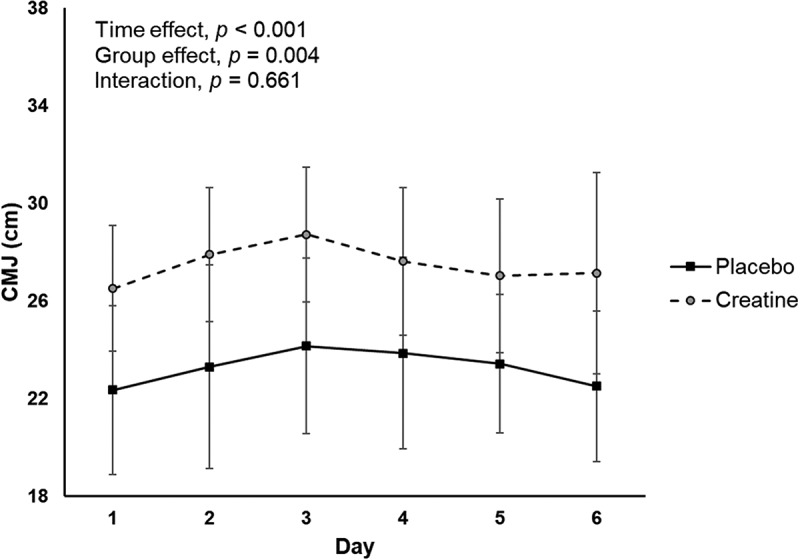
Differences between study groups for countermovement jump (CMJ) height during the training camp.

A significant time effect was observed for the total Hooper index score as well as for the specific items of fatigue and DOMS, which tended to increase over the training camp (Figure 3). However, no significant group by time interaction effect was found for any of these items. Likewise, no significant time or group by time interaction effect was found for the sleep or stress items (Figure 3), and no significant between-group differences were reported for the average Hooper index or its sub-items during the training camp (Table 2).

### 3.2. Body composition

A significant time effect was found for body mass (*p* = 0.001), which tended to increase during the training camp in both groups, and for fat mass (*p* = 0.015), which showed the opposite trend. No significant time effect was however noted for fat-free mass, muscle mass or body water, and no significant group by time interaction effect was found for any body composition variable (Table 3).

**Table 3.** Estimated body composition by group.

Variable	*Creatine* *(n = 11)*		*Placebo* *(n = 12)*		P-value for time effect	P-value for group effect	P-value for group by time interaction effect	Effect size (ηp^2^)
	Baseline	Post	Baseline	Post				
Body mass (kg)	65.8 ± 7.0	66.2 ± 6.6	65.6 ± 6.0	66.5 ± 5.7	0.001	0.981	0.173	0.087
Fat mass (kg)	5.4 ± 2.1	5.2 ± 1.9	6.6 ± 1.9	6.0 ± 1.6	0.015	0.209	0.149	0.096
Fat-free mass (kg)	60.1 ± 6.2	59.9 ± 6.6	59.7 ± 4.7	59.8 ± 5.2	0.662	0.933	0.496	0.022
Muscle mass (kg)	34.5 ± 3.3	34.3 ± 3.6	33.9 ± 2.9	33.9 ± 3.3	0.481	0.707	0.561	0.016
Total body water (%)	44.2 ± 4.2	44.5 ± 4.4	43.7 ± 3.3	43.8 ± 3.7	0.210	0.710	0.388	0.044

Data are shown as mean ± SD.

### 3.3. Performance

A significant time effect was observed for the mean PO attained in the 3-minute and the 6-minute TT, which tended to decrease after the training camp (Table 4). As a result, despite no significant time effect on the remaining TT, a significant time effect was also observed for the estimated CP and W,’ with also tended to decrease after the training camp. No significant group or group by time interaction effect was found, however, for any of the performance variables (Table 4).

**Table 4.** Field-based cycling performance by group.

Outcome	*Creatine* *(n = 11)*		*Placebo* *(n = 12)*		P-value for time effect	P-value for group effect	P-value for group by time interaction effect	Effect size (ηp^2^)
	Baseline	Post	Baseline	Post				
Sprint, PPO (W)	1,131 ± 165	1,094 ± 160	1,055 ± 164	1,050 ± 197	0.315	0.390	0.446	0.028
Sprint, PPO (W/kg)	17.1 ± 1.6	16.6 ± 1.9	16.0 ± 1.6	15.7 ± 2.0	0.135	0.157	0.633	0.011
10-s sprint, mean PO (W)	1,054 ± 157	1,003 ± 167	966 ± 141	973 ± 165	0.270	0.360	0.137	0.102
10-s sprint, mean PO (W/kg)	16.0 ± 1.6	15.2 ± 2.0	14.7 ± 1.4	14.6 ± 1.6	0.106	0.144	0.208	0.075
3-min TT, mean PO (W)	478 ± 29	455 ± 43	476 ± 46	459 ± 52	**0.007**	0.959	0.638	0.011
3-min TT, mean PO (W/kg)	7.3 ± 0.4	6.9 ± 0.6	7.3 ± 0.4	6.9 ± 0.7	**0.001**	0.976	0.751	0.005
6-min TT, mean PO (W)	413 ± 24	387 ± 30	416 ± 45	388 ± 48	**<0.001**	0.879	0.886	0.001
6-min TT, mean PO (W/kg)	6.3 ± 0.4	5.9 ± 0.4	6.3 ± 0.6	5.9 ± 0.7	**<0.001**	0.951	0.764	0.004
12-min TT, mean PO (W)	371 ± 22	365 ± 35	370 ± 36	364 ± 39	0.285	0.937	0.969	0.000
12-min TT, mean PO (W/kg)	5.7 ± 0.4	5.5 ± 0.6	5.7 ± 0.5	5.5 ± 0.6	0.098	0.863	0.829	0.002
CP (W)	338 ± 21	331 ± 30	340 ± 37	329 ± 36	0.065	0.980	0.694	0.008
CP (W/kg)	5.2 ± 0.4	5.0 ± 0.6	5.2 ± 0.6	5.0 ± 0.5	**0.020**	0.901	0.539	0.018
W’ (J)	25476 ± 3741	22091 ± 7424	24986 ± 3722	23237 ± 5062	**0.047**	0.856	0.509	0.021
W’ (J/kg)	389 ± 46	333 ± 101	380 ± 35	350 ± 69	**0.025**	0.832	0.476	0.024

Data are shown as mean ± SD. Abbreviations: CP, critical power; PO, power output; PPO, peak power output; TT, time trial.

## 4. Discussion

The main finding of the present study was that creatine supplementation did not induce beneficial effects on markers of recovery, body composition, or performance during a short-term period of strenuous training loads in professional cyclists.

4.1.

Despite the growing popularity of creatine in endurance sports [19], evidence remains scarce and inconclusive. In fact, a recent meta-analysis including 13 studies found that creatine supplementation does not improve endurance performance [18]. It is worth noting, however, that the indicators used for the assessment of endurance performance might play a major role. For instance, in the aforementioned meta-analysis Fernández-Landa et al. combined different indicators such as the lactate threshold-associated PO, time to exhaustion or VO_2max_ on an incremental test, or performance during a running, swimming or rowing TT [18]. Moreover, different populations were studied, including rugby, handball or soccer players, rowers, and swimmers [18].

Despite the abovementioned results, there is preliminary evidence for a potential ergogenic effect of creatine supplementation in cycling, particularly during high-intensity efforts. Crisafulli et al. reported that creatine supplementation (4 g/day for 6 weeks) improved absolute (i.e. W) PPO (by 4%) and mean PO (by 5%) during a repeated sprint protocol in recreational cyclists compared with a placebo [20]. Interestingly, in the study by Crisafulli et al. the creatine supplement also resulted in a significant increase of body mass (+1.6 kg, *vs* −1 kg in the placebo group) [20], a finding that might be partially accounted for by water retention. However, the authors did not assess relative PO values (i.e. in W/kg). Similarly, Tomcik et al. aimed to examine the effects of creatine supplementation (20 g/day for 5 days followed by a maintenance phase of 3 g/day for 9 days) on performance in a simulated race (120-km TT interspersed with alternating sprints of different durations and a final ride to fatigue at 90% of VO_2_max) in well-trained cyclists and triathletes [21]. Although no supplementation effects were overall found when attending to the whole test, some benefits were observed on the final 4-km sprint. Of note, these benefits on relative PO were significant despite an increase in body mass (+1.5%).

The abovementioned findings might be of relevance, particularly given the stochastic nature of competitive road cycling, including numerous high-intensity intermittent efforts – which also applies to other endurance sports such as mountain biking or triathlon [43,44]. It has been indeed reported that the more successful cyclists spend a larger time at high intensities (e.g. above the FTP or the CP) [45], which suggests that the ability to tolerate such high loads is a major performance determinant. Creatine supplementation might be therefore of potential utility in these contexts, as proposed recently [19]. However, in the present study we failed to find any beneficial effect of creatine supplementation on cycling performance, including short-duration efforts and anaerobic capacity indicators (e.g. 10-second sprint or W’) or longer-duration efforts and aerobic capacity indicators (e.g. 3, 6 and 12-minute TT, or CP). Of note, in the present study no benefits on PO were observed in absolute (W) or relative terms (W/kg), which suggests that the water retention usually associated with creatine supplementation was not the reason underlying the lack of benefits. Indeed, we found a similar body mass gain in both the creatine and placebo groups, which supports that creatine did not promote water retention.

Another major finding of the present study was the lack of effects of creatine supplementation on recovery indicators. Creatine supplementation failed to attenuate the increase in DOMS and perceived fatigue during the training camp, as well as the concomitant impairment in cycling performance. Different mechanisms have been proposed to support the potential of creatine supplementation on recovery, such as its anabolic, antioxidant and anti-inflammatory effects [19]. Moreover, some studies suggest that, due to an increased intracellular water retention and overall cell volume, creatine supplementation might promote a greater capacity for glycogen storage [19]. For instance, in the aforementioned study by Tomcik et al., the combination of creatine and carbohydrates resulted in greater muscle glycogen levels compared with a placebo [21]. These results are in line with previous studies reporting that a high-dose, short-term creatine supplementation protocol similar to the one used in our study (i.e. 20 g/day during 5 days) combined with carbohydrate ingestion resulted in higher muscle glycogen levels (by 18 to 53%) compared with carbohydrates alone [46,47]. Unfortunately, markers of oxidative stress, inflammation, or muscle glycogen levels were not measured in the present study.

The characteristics of the creatine supplementation protocol used in the present study might explain, at least partly, our results. Different studies show that high-dose short-term creatine supplementation (e.g. 20 g during 5–6 days) is effective to increase muscle creatine content [3,4,8,17,48]. However, some studies have reported no significant benefits of short-term creatine supplementation on performance [7,8]. For instance, Finn et al. reported that although the ingestion of 20 g of creatine during 5 days was effective for increasing intramuscular creatine content, the benefits observed on PPO and fatigue resistance compared to a placebo during a repeated sprint protocol did not reach statistical significance (*p*=0.05 and *p* = 0.08, respectively) [8]. Notwithstanding, most evidence shows that short-term creatine supplementation is effective for enhancing performance, as reflected by increases in muscle strength, fatigue resistance or PO during a single effort (e.g. 100-m running sprint, Wingate anaerobic test) or consecutive sprints [9–17]. For instance, a recent meta-analysis of 14 randomized controlled trials concluded that short-term creatine supplementation increases mean PO during repeated sprints [9]. It is worth noting, however, that most studies applying a short-term protocol have used split-dosing (usually 4–5 doses of 4–5 g) through the day [2]. Here, however, we provided a single daily creatine dose of 20 g for practical reasons, as this enabled us to monitor its intake, and we ensured that the whole supplement was taken after exercise. Regarding the latter, although scarce and mixed evidence exists [49], some reports suggest that ingesting creatine following exercise might provide additional benefits on performance and body composition [50–53]. It can be hypothesized, however, that a longer-term protocol with a lower (or split) creatine dose could have been better tolerated by the athletes and potentially resulted in greater performance gains.

Some limitations of the present study should be noted, such as the relatively small sample size, which might be justifiable given the high performance level of the recruited participants. Moreover, although we used a parallel-group design (which avoids the limitations of a potential carryover effect), a crossover design could have reduced the potential confounding effect of between-individual variability. Another limitation is the fact that we did not standardize or monitored participants’ diet during the whole training camp, although we assessed their dietary intake during at least two days to minimize this confounding factor. Unfortunately, we could not assess muscle creatine levels using biopsies, as per ethical constraints. However, high-dose short-term creatine supplementation protocols such as that applied here (20 g/day during 4–6 days) have previously proven effective for increasing muscle creatine levels [3,4,8,17,48]. Similarly, the fact of not having assessed muscle glycogen levels or other molecular indicators (e.g. markers of anabolism, inflammation, or oxidative stress) can also be considered a study limitation. Finally, the use of more sensitive or complex methods (e.g. dual X-ray absorptiometry or skinfold measurements instead of bioimpedance, or including a laboratory test or a time to exhaustion test as a performance measure) could have provided greater insights on the study question. In turn, the fact of having performed a nutritional intervention in highly-trained cyclists and the variety of assessed outcomes can be considered major strengths of the study.

## 5. Conclusions

High-dose, short-term creatine supplementation (20 g/day in a single intake for 7 days) did not induce significant effects on markers of recovery, body composition, or performance during a training camp in professional cyclists, which suggests that this protocol might not be beneficial during periods of strenuous training loads. Future studies should confirm whether creatine supplementation exerts beneficial effects on endurance sports, and particularly whether a longer-term protocol with a lower (or split) creatine dose could result in greater benefits on recovery or performance during periods of high training loads.

## Supplementary Material

Supplemental Material
