# Supplementary material for: High-dose short-term creatine supplementation without beneficial effects in professional cyclists: a randomized controlled trial
Source: J Int Soc Sports Nutr. 2024 Apr 12;21(1):2340574. doi: 10.1080/15502783.2024.2340574 (PMC11018046; doi:10.1080/15502783.2024.2340574)
Supplement: Supplemental Material [file RSSN_A_2340574_SM8993.docx]

|  | Day 1 | Day 2 | Day 3 | Day 4 | Day 5 | Day 6 |
| --- | --- | --- | --- | --- | --- | --- |
| Duration (hours) | 3 | 3.5 | 4 | 6 | 6 | 2.5 |
| Target intensity (IF) | ~0.65 | ~0.75 | ~0.70 | ~0.70 | ~0.75 | ~0.55 |
| Objective | Easy ride | 3 x 15 min uphill (105-110% of FTP). Recovery: 20 min. | Technical-tactical work (e.g., side wind, sprint throws, long relays, short relays, rhythm work towards a leader) | Volume training with ~3000 m of elevation gain. | “Race Day”. Controlled ride with some free segments where cyclists show their maximum physical potential. | Easy ride |

**Supplemental Table.** Example of the prescribed training sessions during the training camp.

Abbreviations: IF, intensity factor; FTP, functional threshold power.
